# Employing whole genome mapping for optimal *de novo* assembly of bacterial genomes

**DOI:** 10.1186/1756-0500-7-484

**Published:** 2014-07-30

**Authors:** Basil Britto Xavier, Julia Sabirova, Moons Pieter, Jean-Pierre Hernalsteens, Henri de Greve, Herman Goossens, Surbhi Malhotra-Kumar

**Affiliations:** 1 Department of Medical Microbiology, Vaccine & Infectious Disease Institute, Universiteit Antwerpen, Antwerp, Belgium; 2 Viral Genetics Research Group, Vrije Universiteit Brussel, Brussels, Belgium; 3 Structural Biology Brussels, Flanders Institute for Biotechnology (VIB), Vrije Universiteit Brussel, Brussels, Belgium

**Keywords:** *De-novo* assembly, Whole genome mapping, *k-mer*, Microbial genomes, de bruijn graph, SPAdes and Velvet

## Abstract

**Background:**

*De novo* genome assembly can be challenging due to inherent properties of the reads, even when using current state-of-the-art assembly tools based on de Bruijn graphs. Often users are not bio-informaticians and, in a black box approach, utilise assembly parameters such as contig length and N50 to generate whole genome sequences, potentially resulting in mis-assemblies.

**Findings:**

Utilising several assembly tools based on de Bruijn graphs like Velvet, SPAdes and IDBA, we demonstrate that at the optimal N50, mis-assemblies do occur, even when using the multi-*k-mer* approaches of SPAdes and IDBA. We demonstrate that whole genome mapping can be used to identify these mis-assemblies and can guide the selection of the best *k-mer* size which yields the highest N50 without mis-assemblies.

**Conclusions:**

We demonstrate the utility of whole genome mapping (WGM) as a tool to identify mis-assemblies and to guide *k-mer* selection and higher quality *de novo* genome assembly of bacterial genomes.

## Findings

Genome assembly is often a primary step in the process of yielding results that lead to interpretation of biological data and hence sub-optimally assembled genomes might lead to faulty conclusions [1]. Factors causing such low quality genome assembly include sequence quality, presence of repetitive sequences, base composition, size and low genome coverage [2,3], all of which complicate downstream data analysis using the available tools [4]. Currently, *de novo* assemblers based on de Bruijn graph are considered to yield the best results provided sufficient sequence quality and coverage are achieved. Such assembly tools based on de Bruijn graph algorithms, like Velvet [5] and SPAdes [6] use *k-mers* as building blocks, but as most users are not bio-informaticians, these tools are often considered as an encrypted black box with the quality of the assembly usually determined by statistics parameters such as the N50 and the size and number of contigs or scaffolds produced by the assemblers [7]. However, the choice of the *k-mer* size is crucial as too low or too high *k-mer* sizes lead to sub-optimal assemblies. Indeed, low quality reads might produce false positive vertices, repeats lead to branching, while an uneven distribution of the reads results in gaps. The use of smaller *k-mers* reduces the problem associated with low quality reads and their uneven distribution, while larger *k-mer* sizes help to bridge repeat regions decreasing the branching problem [8]. In a balancing exercise, various *k-mer* sizes are usually selected, evaluating optimization by aiming for high N50 values and long, but fewer contigs. Whole Genome Mapping (WGM; Opgen Inc, Gaithersburg, MD, USA) is a relatively novel technique that generates high-resolution restriction maps of a genome based on the alignment of single DNA molecules cut with restriction enzymes and ordered with high resolution and accuracy [9]. WGM was proven helpful in mapping *de novo* assembled contigs against previously sequenced related genomes [10].

**Figure 1:**
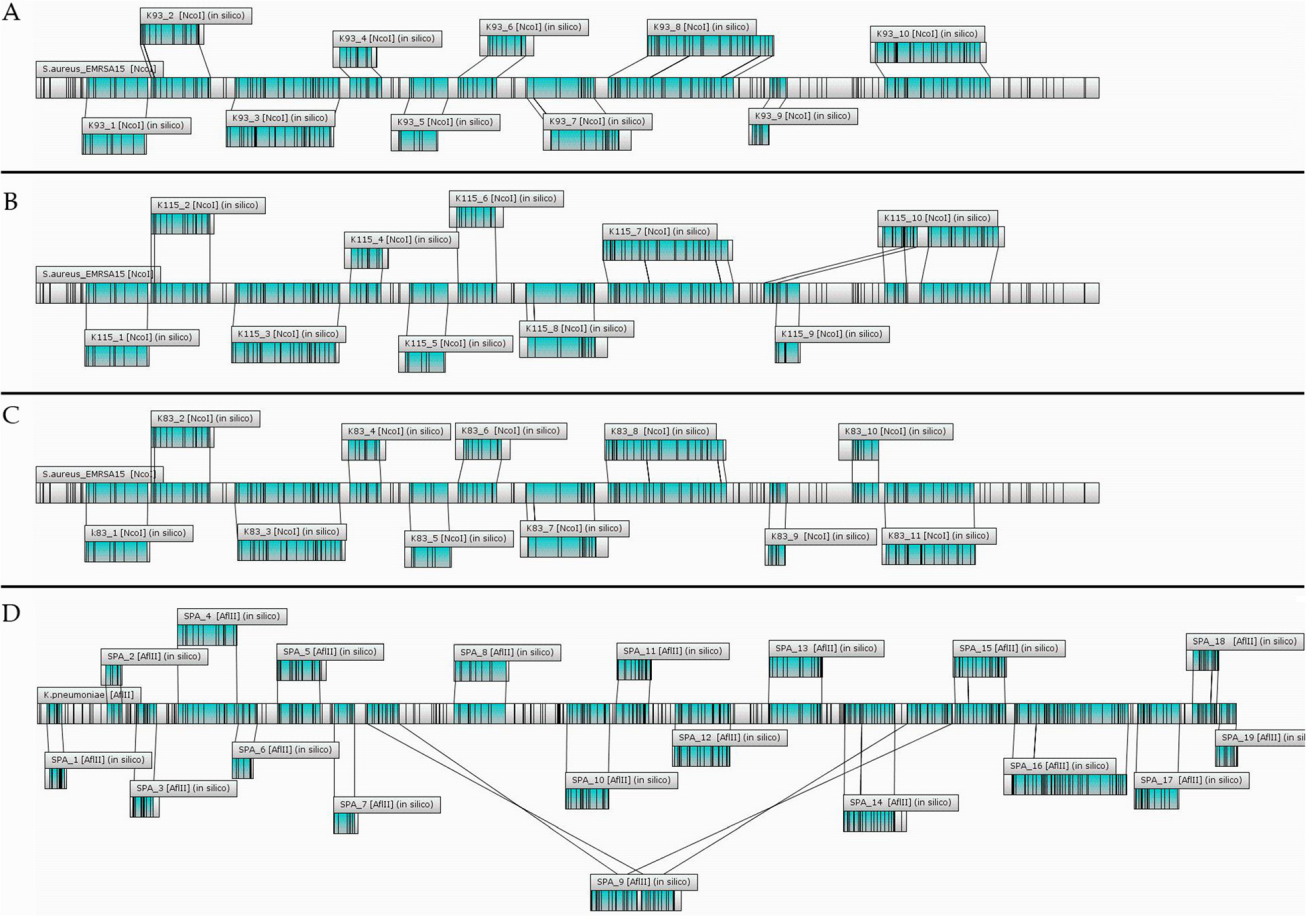
**Alignment of contigs to the corresponding whole genome map: A) Velvet derived assembly using *k-mer *** **size 93, revealing no mis-assemblies; B) Velvet derived assembly using *k-mer *** **size 115, corresponding to the highest N50, but revealing mis-assemblies; C: SPAdes derived assembly using a multi-*k-mer *** **approach up to k-mer size 83, yielding the optimal N50 for this sequence and showing no mis-assemblies; D: SPAdes derived assembly using a multi-*k-mer *** **approach up to k-mer size 77, yielding the optimal N50 for this sequence, but showing mis-assemblies.**

In this paper, we evaluated the utility of WGM for proper *k-mer* size selection and for optimization of parameters for *de novo* genome assembly. The whole genome sequence of a methicillin-resistant *Staphylococcus aureus* (MRSA) strain (E-MRSA15-CC22-SCC*mec*IV) was generated on an Illumina HiSeq-2000 via 2X150b paired end sequencing [11]. Reads were *de novo* assembled using Velvet, SPAdes and IDBA-UD employing a range of *k-mers*. Velvet, using a single *k-mer* approach ranging from *k-mer* size 81 to 123 showed an initial increase in N50 (until *k-mer* size 115) and longest contig size and a concomitant decrease in the total number of contigs with increasing *k-mer* size with both these parameters positively influencing the assembly outcome (Table 1). Ensuing, using WGM, a whole genome map of *S. aureus* EMRSA-15 was generated which, using MapSolver, was aligned with the assembly files corresponding to different *k-mer* sizes. Although the percentage of the genome covered by contigs increased with increasing *k-mer* size, a mis-assembly (spanning 119 kb) was identified for the mapped contigs (>40 kb) for higher *k-mer* sizes (Table 1), revealing the best (without mis-assemblies) assembly was actually obtained using a *k-mer* size of 93 despite a higher N50 and fewer contigs as when for example utilizing a *k-mer* size of 115 (Table 1, Figure 1A, B). In contrast, SPAdes, which allows to combine a range of *k-mer* sizes in a multi-*k-mer* approach did not yield any mis-assemblies on this sequence for the N50 based best assemblies (Figure 1C). The same was true for IDBA, which similarly utilizes an iterative process including multiple *k-mer* sizes, while removing assembled sequences in subsequent rounds of analysis.

**Table 1:** **Assembly statistics of Velvet applied on *Staphylococcus aureus *** **(MRSA) strain E-MRSA15-CC22-SCC *mec *** **IV showing an increase in contig size and N50 when using higher *k-mer *** **sizes, but revealing a mis-assembly starting from *k-mer *** **size 97 using whole genome mapping**

**K-mer size**	**N50**	**Total number of contigs**	**Longest contig size**	Mis-assemblies on mapped contigs*	**Approx. nts involved in mis-assemblies**
**Velvet**					
81	162295	40	340060	1 (10)	122303
83	170447	38	351373	1 (9)	122303
85	170449	37	351321	0 (10)	
87	173763	33	351326	0 (10)	
89	173765	33	351394	0 (10)	
91	173767	33	351330	0 (10)	
93	173769	35	340092	0 (10)	
97	175770	33	365247	1 (9)	130273
99	175776	33	365260	1 (10)	130273
101	187438	32	365623	1 (9)	130273
103	187448	32	365625	1 (9)	130273
105	187458	32	365638	1 (9)	130273
107	187465	33	365647	1 (9)	130273
109	212189	32	365656	1 (9)	130273
111	212287	33	349286	2 (8)	93632 & 153207
113	212292	34	349288	1 (10)	93634
115	212294	34	349290	1 (10)	118928
117	174074	35	349419	1 (11)	93634
119	174076	35	349423	1 (11)	93638
121	170642	37	349435	0 (11)	
123	170654	38	340456	0 (10)	

*Number of mapped contigs indicated between brackets.

The general applicability of these results was investigated using two additional, similarly obtained, *S. aureus* sequences [UA-S391(accession # CP007690) and Mu50-CC5-SCC*mec*II (ATCC700699; previously sequenced and available under accession # NC_002758], again revealing mis-assemblies for Velvet at the highest N50 values, while error free assemblies could be obtained for lower *k-mer* sizes (data not shown). In addition, the sequence of *Klebsiella pneumoniae* ST258 was similarly generated using Illumina HiSeq-2000 via 2X150b paired end sequencing and was assembled using all three assembly tools. In this case, apart from mis-assemblies seen for Velvet, also SPAdes and IDBA were shown to produce mis-assemblies for certain *k-mer* sizes (Figure 1D), further demonstrating the potential of WGM to identify mis-assemblies, even for assemblers utilizing multi-*k-mer* approaches (Additional file 1: Table S2).

## Conclusion

Genome assembly based on de Bruijn graphs potentially yields mis-assemblies when only considering standard parameters such as total number and length of the contigs and N50. However, Whole Genome Mapping provides a powerful tool to identify such mis-assemblies and to select the optimal *k-mer* sizes to produce optimally assembled genomes. Despite of its additional cost, the biological need for error-free and complete genomes makes WGM an indispensable technique during the process of genome assembly and its validation.

## Competing interests

The authors declare that they have no competing interests.

## Authors’ contributions

BBX participated in the design of the study and performed the data analysis; JS participated in the design of the study and was involved in whole genome mapping; PM participated in conceiving and drafting the manuscript and performed the image processing; JPH and HdG provided the strains and were involved in sequencing for this study; HG and SMK participated in conceiving the study and revised the manuscript. All authors read and approved the final manuscript.

## Supplementary Material

Additional file 1: Table S2 Assembly statistics of Velvet, SPAdes and IDBA-UD applied on *Staphylococcus aureus* (MRSA) strain E-MRSA15-CC22-SCC*mec*IV and assembly statistics of Velvet for *Klebsiella pneumoniae* showing an initial increase in contig size and N50 when using higher *k-mer* sizes, but revealing mis-assemblies associated with higher k-mer sizes in some cases. Click here for file
