# Supplementary material for: Employing whole genome mapping for optimal de novo assembly of bacterial genomes
Source: BMC Res Notes. 2014 Jul 30;7:484. doi: 10.1186/1756-0500-7-484 (PMC4118782; doi:10.1186/1756-0500-7-484)
Supplement: Additional file 1: Table S2 — Assembly statistics of Velvet, SPAdes and IDBA-UD applied on Staphylococcus aureus (MRSA) strain E-MRSA15-CC22-SCCmecIV and assembly statistics of Velvet for Klebsiella pneumoniae showing an initial increase in contig size and N50 when using higher k-mer sizes, but revealing mis-assemblies associated with higher k-mer sizes in some cases. [file 1756-0500-7-484-S1.docx]

| K-mer Size | N50 | Total number of contigs | Longest contig size | Mis-assemblies on mapped contigs |
| --- | --- | --- | --- | --- |
| Velvet on *Staphylococcus* *aureus* | | | | |
| 81 | 162295 | 40 | 340060 | 1(10) |
| 83 | 170447 | 38 | 351373 | 1 (9) |
| 85 | 170449 | 37 | 351321 | 0 (10) |
| 87 | 173763 | 33 | 351326 | 0 (10) |
| 89 | 173765 | 33 | 351394 | 0 (10) |
| 91 | 173767 | 33 | 351330 | 0 (10) |
| 93 | 173769 | 35 | 340092 | **0 (10)** |
| 97 | 175770 | 33 | 365247 | 1 (9) |
| 99 | 175776 | 33 | 365260 | 1 (9) |
| 101 | 187438 | 32 | 365623 | 1 (9) |
| 103 | 187448 | 32 | 365625 | 1 (9) |
| Spades on *Staphylococcus* *aureus* | | | | |
| 75 | 127316 | 107 | 326292 | 0 (11) |
| 77 | 170615 | 101 | 326296 | 0 (11) |
| 79 | 170619 | 91 | 326300 | 0 (11) |
| 81 | 170623 | 89 | 326304 | 0 (11) |
| 83 | 170627 | 89 | 326308 | 0 (11) |
| 85 | 170631 | 86 | 326312 | 0 (11) |
| 87 | 146398 | 83 | 326400 | 0 (11) |
| 89 | 146401 | 80 | 327176 | 0 (11) |
| 91 | 146405 | 72 | 327092 | 0 (11) |
| 93 | 146409 | 75 | 327094 | 0 (11) |
| 97 | 146417 | 76 | 327098 | 0 (11) |
| 99 | 146421 | 74 | 327100 | 0 (11) |
| 101 | 146425 | 78 | 327102 | 0 (11) |
| IDBA-UD on *Staphylococcus* *aureus* | | | | |
| 97 | 73691 | 342 | 286938 | 0 (8) |
| 99 | 78050 | 137 | 286942 | 0 (9) |
| 101 | 86931 | 266 | 286946 | 0 (11) |
| 103 | 86935 | 253 | 286950 | 0 (11) |
| Velvet on *Klebsiella pneumoniae* | | | | |
| 75 | 160441 | 147 | 713377 | 0 (21) |
| 77 | 193706 | 146 | 713416 | 0 (20) |
| 79 | 143602 | 140 | 802887 | 0 (19) |
| 81 | 270037 | 143 | 713577 | 1^**^ (18) |
| 83 | 193711 | 151 | 713924 | 1^**^ (18) |
| 85 | 225886 | 148 | 713989 | 1^**^(18) |
| 87 | 203660 | 148 | 803407 | 1^**^ (18) |
| 89 | 193696 | 158 | 713922 | 1^*^ (19) |

^**^386153 nt involved in mis-assembly. ^*^ 181195 nt involved in mis-assembly

Additional file 1: Table S2 Assembly statistics of Velvet, SPAdes and IDBA-UD applied on Staphylococcus aureus (MRSA) strain E-MRSA15-CC22-SCCmecIV and assembly statistics of Velvet for *Klebsiella pneumoniae* showing an initial increase in contig size and N50 when using higher k-mer sizes, but revealing mis-assemblies associated with higher k-mer sizes in some cases.
